# Epidemiology and risk factors of urolithiasis: insights from SKIPOGH, a population-based cohort study in Switzerland

**DOI:** 10.1093/ckj/sfag002

**Published:** 2026-01-07

**Authors:** Kevin Stritt, Maude Plouvin, Sandrine Estoppey Younes, Belen Ponte, Daniel Ackermann, Daniel G Fuster, Oliver Bonny, Beat Roth, Murielle Bochud, Menno Pruijm

**Affiliations:** Department of Urology, University Hospital Lausanne (CHUV), University of Lausanne, Lausanne, Switzerland; Department of Urology, Inselspital, Bern University Hospital, University of Bern, Bern, Switzerland; Department of Urology, University Hospital Lausanne (CHUV), University of Lausanne, Lausanne, Switzerland; Department of Epidemiology and Health Systems, Unisanté, University Center for Primary Care and Public Health & University of Lausanne, Lausanne, Switzerland; Service de Néphrologie et Hypertension, Hôpitaux Universitaires de Genève, Geneva, Switzerland; Department of Nephrology and Hypertension, Inselspital, Bern University Hospital, University of Bern, Bern, Switzerland; Department of Nephrology and Hypertension, Inselspital, Bern University Hospital, University of Bern, Bern, Switzerland; Service of Nephrology, Fribourg State Hospital, Fribourg, Switzerland; Department of Urology, Inselspital, Bern University Hospital, University of Bern, Bern, Switzerland; Department of Epidemiology and Health Systems, Unisanté, University Center for Primary Care and Public Health & University of Lausanne, Lausanne, Switzerland; Service of Nephrology, University Hospital Lausanne (CHUV), University of Lausanne, Lausanne, Switzerland

**Keywords:** cohort study, prevalence, risk factors, urolithiasis

## Abstract

**Background:**

Kidney stones represent a growing global health concern, with a lifetime prevalence estimated at 7%–13% in North America and 1%–5% in Asia, but European data are scarce. We assessed the prevalences and incidence of kidney stones in the Swiss adult population and identified associated factors.

**Methods:**

The Swiss Kidney Project on Genes in Hypertension (SKIPOGH) is a multicenter cohort including 1128 participants recruited from the general population of Lausanne, Geneva and Bern (2009–12). All underwent renal ultrasound at baseline and completed a standardized questionnaire. Predefined demographic, lifestyle and clinical variables included age, sex, BMI, education, smoking, physical activity, hypertension, diabetes, history of kidney stones, laboratory and urinary parameters. A follow-up visit was performed 3 years later. Logistic regression analysis was performed to identify factors associated with kidney stone prevalence at baseline and 3-year incidence of newly formed stones.

**Results:**

Ultrasound-detected kidney stones were present in 5.6% (6.1% men and 5.1% women). The 3-year incidence of new stones was 4.3% (4.1% men, 4.6% women). In multivariable logistic regression analysis, diabetes mellitus [odds ratio (OR) 2.93, 95% confidence interval (CI) 1.05–8.21, *P* = .04], a family history of kidney stones (OR 9.96, 95% CI 4.53–21.91, *P* < .01) and higher serum creatinine (OR 1.02 per µmol/L, 95% CI 1.00–1.04, *P* = .02) were associated with the prevalence of kidney stones. Active smoking (OR 2.49, 95% CI 1.07–5.78, *P* = .03), lower physical activity score (OR 0.82, 95% CI 0.67–1.00, *P* = .05) and a personal history of kidney stones (OR 33.0, 95% CI 12.4–87.6, *P* < .01) were associated with the 3-year incidence of kidney stones.

**Conclusion:**

In this Swiss cohort, the prevalence of kidney stones was lower than that reported in North America but higher than in Asian populations. Diabetes mellitus, family history of kidney stones were the strongest risk factors for prevalent stones, while a personal history of kidney stones and low physical activity were predictors of incident stone formation. These data will help to inform prevention strategies.

KEY LEARNING POINTS **What was known:**

- Kidney stone disease is a common and recurrent condition with increasing prevalence worldwide, but there are limited contemporary data from European populations.
- Risk factors such as obesity, diabetes and dietary habits are well established in North American and Asian cohorts.
- Data on the incidence and determinants of kidney stones in the general Swiss population were lacking.

**This study adds:**

- This is the first population-based study to estimate both the prevalence and 3-year incidence of kidney stones in Switzerland.
- Diabetes, family history of stones and higher serum creatinine were identified as independent predictors of prevalent stones.
- Low physical activity, smoking and previous history of stones were key risk factors for new stone formation.

**Potential impact:**

- These findings highlight modifiable lifestyle and metabolic risk factors that can inform targeted prevention strategies in the general population.
- The results may support clinicians in risk stratification and counseling for stone prevention.
- The study provides an epidemiological benchmark for monitoring kidney stone trends in Europe.

## Introduction

Urolithiasis is a common and recurrent condition with significant implications for public health and healthcare systems worldwide. Its prevalence ranges from 7% to 13% in North America, and 1% to 5% in Asia, reflecting regional variations in screening, environmental exposure, dietary habits and genetic predisposition [1, 2]. In addition to causing acute pain and long-term renal complications, urolithiasis has been recognized as a systemic condition. Several studies have demonstrated its associations with chronic metabolic disorders, such as diabetes mellitus and hypertension [3, 4], as well as an elevated risk of cardiovascular disease and bone diseases [5].

Epidemiological evidence suggests that kidney stone formation follows a characteristic age-related distribution, peaking between the ages of 40 and 60 years [6]. Furthermore, male sex, obesity and low urine volume are established risk factors for stone formation, while dietary factors such as high sodium and animal protein intake may also contribute [7, 8]. Despite these known associations, the strength and independence of these risk factors vary across populations, and comprehensive data from European population-based cohorts remain limited, with prevalence estimates between 5% and 9% [1]. Moreover, population-based studies simultaneously combining different detection methods—such as questionnaires and imaging modalities—to assess kidney stone prevalence and associated risk factors remain scarce.

Leveraging data from the Swiss Kidney Project on Genes in Hypertension (SKIPOGH)—a family- and general population–based cohort—we aimed to assess the prevalence and incidence of kidney stones and to identify independent risk factors for their formation in a representative Swiss adult population. By clarifying the most relevant clinical, demographic and biochemical predictors, this study seeks to inform prevention strategies and improve risk stratification, particularly in aging individuals and those with metabolic comorbidities [9, 10].

## Materials and methods

### Study design and population

This study used data from SKIPOGH, a multicenter, family- and general population–based cohort designed to examine genetic and environmental determinants of blood pressure and kidney function [11]. Participants were recruited between October 2009 and April 2012 from three regions in Switzerland: Lausanne, Geneva and Bern. Probands were identified through population-based recruitment using random sampling of the general population in each center. Inclusion criteria were: age ≥18 years, European descent, signed informed consent and having at least one first-degree family member willing to participate; only these first-degree relatives were recruited as part of the family component of the cohort. A total of 1128 adult participants were included at baseline in this cohort study. All individuals filled in a questionnaire and underwent standardized clinical assessments, including anthropometric measurements, laboratory testing and renal ultrasound by trained nephrologists to evaluate kidney morphology and detect the presence of urolithiasis. A follow-up visit repeating the same exams took place 3 years later, between 2012 and 2016, in 87% of participants [11].

### Assessment of kidney stones

Trained nephrologists performed all the examinations using renal doppler ultrasounds following a standardized procedure [12]. Kidney stones were identified as hyperechogenic lesions with a posterior acoustic shadow, the presence of a “twinkling” artifact on color Doppler or indirect signs of obstruction [13]. The presence, size and anatomical location of stones were documented (left, right or bilateral). Intraparenchymal calcifications—which are not located within the pyelocaliceal system—were excluded from the analysis, as they are not considered true stones.

Additionally, participants completed a standardized questionnaire on their history of kidney stones and previous symptomatic events. To evaluate point prevalence, we considered the presence of kidney stones detected on ultrasound at baseline. Lifetime prevalence was defined as a positive self-reported history of kidney stones and/or stones detected on baseline ultrasound. For the 3-year incidence, we identified new cases of kidney stones on follow-up ultrasound among participants, irrespective of baseline status.

### Covariates and risk factors assessment

A broad set of demographic, lifestyle, clinical and laboratory variables was evaluated for their association with urolithiasis. These included:

- Demographic variables: age and sex
- Lifestyle factors: smoking status (never, former, current) and physical activity score (1–10 scale, encompassing occupational, leisure and sports-related effort; 1 = sedentary, 10 = heavy labor)
- Anthropometric and metabolic indicators: body mass index (BMI), obesity (BMI ≥30 kg/m²), diabetes mellitus (self-reported physician diagnosis, use of antidiabetic medication or fasting glucose ≥7.0 mmol/L) and hypertension (self-reported diagnosis, use of antihypertensive medication or systolic/diastolic blood pressure ≥140/90 mmHg)
- Blood biomarkers: serum corrected calcium, total calcium, uric acid, magnesium, bicarbonate, creatinine and estimated glomerular filtration rate (eGFR)
- Twenty-four-hour urinary parameters: urine volume, excretion of calcium, sodium, magnesium, phosphate, uric acid and urinary pH
- Clinical history: prior diagnosis of kidney stones, self-reported history of kidney stones and first-degree family history of kidney stones were also included, particularly in modeling stone incidence during follow-up

All covariates were collected in accordance with SKIPOGH standardized protocols [12].

### Statistical analysis

Descriptive statistics were used to summarize baseline characteristics. Continuous variables were expressed as medians with interquartile ranges (IQRs), and categorical variables as frequencies and percentages. Mixed-effects logistic regression analyses were performed to account for the family structure of the SKIPOGH cohort, with family included as a random effect and all other covariates treated as fixed effects. Associations were assessed for (i) the point prevalence of kidney stones at baseline and (ii) the 3-year incidence of new stone formation.

Variables with a *P*-value <.10 in univariable analyses were entered into mixed multivariable logistic regression models to identify independent factors associated with the incidence and prevalence of kidney stones. The predefined variables age, sex, diabetes mellitus, BMI, hypertension, education level, family or personal history of stones, 24-h urine volume and urinary sodium excretion were also retained in the adjusted models irrespective of their univariable significance.

Results are reported as odds ratios (ORs) with 95% confidence intervals (CIs), and statistical significance was defined as a two-sided *P*-value <.05. All analyses were conducted using Stata version 18.0 (StataCorp LLC, College Station, TX, USA).

## Results

Baseline demographic characteristics and laboratory parameters are summarized in Table 1. Of the 1128 participants included, 540 (47.8%) were men and 588 (52.2%) women, with a median age of 48 years (IQR 32–61) and median BMI of 24 kg/m² (IQR 22–27). Obesity (BMI ≥30 kg/m²) was present in 13.0%. Most participants had completed secondary (51.5%) or higher education (36.4%). Regular physical activity was reported by 59.8%. Smoking status was distributed as 45.0% never, 30.9% former and 24.1% current smokers. Hypertension and diabetes mellitus were reported in 24.6% and 3.8% of participants, respectively. At baseline, a previous history of kidney stones was present in 2.5% and a family history in 3.7%. Median serum creatinine was 72 µmol/L (IQR 64–82), and 3.5% had eGFR ≤60 mL/min/1.73 m². Median 24-h urine volume was 1571 mL (IQR 1113–2126).

**Table 1:** Baseline characteristics of the study population.

Characteristics	Total (*N* = 1128)
**Demographics**	
Sex, *n* (%)	
Male	540 (47.8)
Female	588 (52.2)
Age, years, median (IQR)	48 (32–61)
BMI, kg/m², median (IQR)	24 (22–27)
Obesity, *n* (%)	146 (13.0)
Education level, *n* (%)	
No diploma	31 (2.8)
Mandatory school	103 (9.3)
Secondary education	570 (51.5)
Superior education	180 (16.3)
University	222 (20.1)
**Lifestyle factors**	
Smoking status, *n* (%)	
Never smoker	508 (45.0)
Former smoker	349 (30.9)
Current smoker	272 (24.1)
Physical activity score, median (IQR)	5 (4–6)
Clinical characteristics, *n* (%)	
Hypertension	278 (24.6)
Diabetes mellitus	43 (3.8)
Personal history of kidney stones	28 (2.5)
Family history of kidney stones	42 (3.7)
Laboratory and urinary characteristics	
Serum corrected calcium, mmol/L, median (IQR)	2 (2–2)
Serum total calcium, mmol/L, median (IQR)	2 (2–2)
Serum uric acid, µmol/L, median (IQR)	301 (254–358)
Serum creatinine, µmol/L, median (IQR)	72 (64–82)
eGFR ≤60 mL/min/1.73 m², *n* (%)	39 (3.5)
24-h urine volume, mL, median (IQR)	1571 (1113–2126)
Urinary calcium (24 h), mmol, median (IQR)	4 (2–5)
Urinary sodium (24 h), mmol, median (IQR)	135 (100–176)
Urinary magnesium (24 h), mmol, median (IQR)	4 (3–5)
Urinary phosphate (24 h), mmol, median (IQR)	25 (20–32)
Urinary uric acid (24 h), µmol, median (IQR)	1929 (1130–2980)
Urinary pH, median (IQR)	6 (5–7)

Data are presented as *n* (%) for categorical variables and as median (IQR) for continuous variables.

Obesity is defined as BMI ≥30 kg/m². Diabetes mellitus is defined as self-reported physician diagnosis or use of antidiabetic medication. Hypertension is defined as self-reported physician diagnosis, use of antihypertensive medication or measured blood pressure ≥140/90 mmHg at baseline. Physical activity score corresponds to self-reported daily physical effort on a 1–10 scale, including work, sports and leisure activities.

**Table 2:** Prevalence and incidence of kidney stones in the SKIPOGH cohort.

Measure	Definition	Total (1128)^a^	Men (540)^a^	Women (588)^a^
Baseline prevalence	Kidney stones detected by US at baseline	63 (5.6)	33 (6.1)	30 (5.1)
Personal history of kidney stone	Self-reported history of kidney stones at baseline	28 (2.5)	12 (2.2)	16 (2.7)
Combined lifetime prevalence	US and/or self-reported kidney stone at baseline	88 (7.8)	43 (8.0)	45 (7.6)
Asymptomatic stones	Stones detected by US without self-reported history	58 (5.1)	31 (5.7)	27 (4.6)
3-year incidence	New stones at follow-up detected by US	49 (4.3)	22 (4.1)	27 (4.6)

^a^Data are presented as *n* (%).

Point prevalence = kidney stones detected by ultrasound at baseline. Personal history of kidney stone = self-reported history of kidney stone at baseline. Combined lifetime prevalence = ultrasound and/or self-report kidney stone. Incidence = new stones detected at follow-up among participants stone-free at baseline (US and/or self-report).

US, ultrasound.

### Stone prevalence at baseline

At baseline, kidney stones were detected by ultrasound in 63 participants, yielding a point prevalence of 5.6% (6.1% in men and 5.1% in women). A self-reported history of kidney stones was recorded in 28 participants (2.5%), leading to a lifetime prevalence at baseline of 7.8%, with similar proportions in men (8.0%) and women (7.6%). Notably, 58 participants (5.1%) had kidney stones detected at ultrasound in the absence of any self-reported history of nephrolithiasis and without symptoms. When stratified by age, kidney stone prevalence was lower in young adults, peaked in middle age and declined slightly in older participants, consistent with a bell-shaped distribution (Fig. 2).

**Figure 1:**
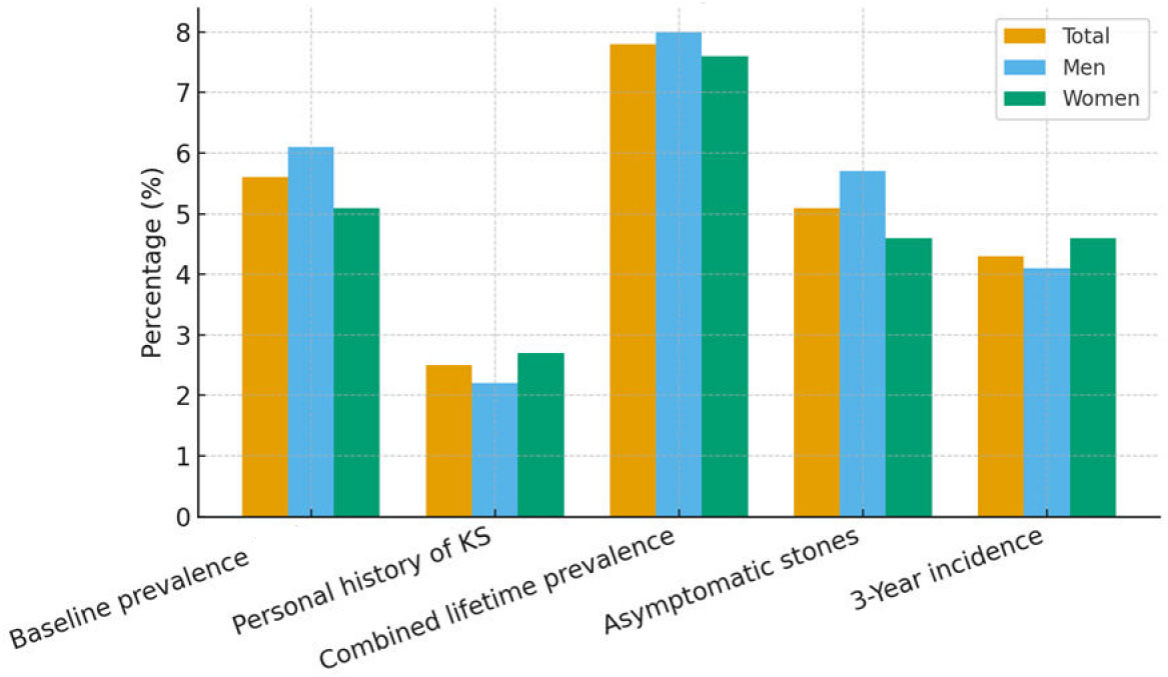
Prevalence and 3-year incidence of kidney stones in the SKIPOGH cohort. Bars represent the prevalence and incidence of kidney stones in the SKIPOGH cohort, stratified by sex. Baseline prevalence was defined as stones detected by ultrasound at baseline. Personal history of kidney stone refers to self-reported kidney stone history at baseline. Combined lifetime prevalence includes either ultrasound-detected or self-reported stones. Asymptomatic stones were defined as ultrasound-detected stones without self-reported history. Three-year incidence corresponds to new cases detected at follow-up among participants stone-free at baseline. KS, kidney stone.

**Figure 2:**
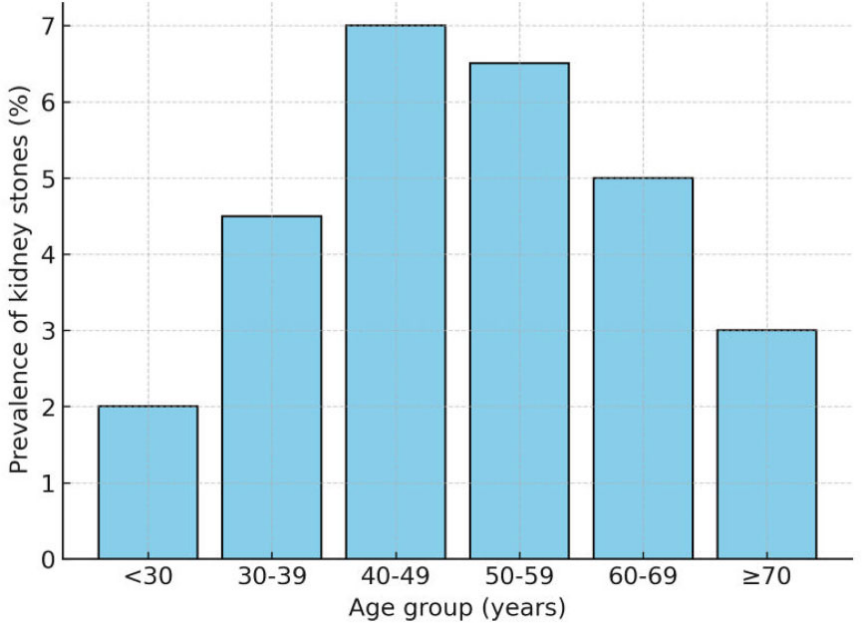
Age distribution of kidney stone point prevalence. Age groups were defined in 10-year intervals. Point prevalence was calculated as the proportion of participants with ultrasound-confirmed stones within each group.

Among the 63 participants with kidney stones detected at baseline, the median number of stones per patient was 1 (IQR 1–2; range 1–5), and the median largest stone diameter was 5 mm (IQR 4–7; range 2–15 mm). In terms of anatomical distribution, stones were mostly located in the lower pole (42.9%), followed by the middle calyx (38.1%) and the upper pole (19.0%).

In univariable logistic regression, diabetes mellitus (OR 2.81, 95% CI 1.01–7.81, *P* = .05), a family history of kidney stones (OR 9.89, 95% CI 4.52–21.61, *P* < .01) and higher serum creatinine (OR 1.02 per µmol/L, 95% CI 1.00–1.04, *P* = .02) were associated with stone prevalence.

In the mixed multivariable logistic regression model, we included the predefined variables age, sex, diabetes, BMI, hypertension, education level, family or personal history of stones, 24-h urine volume and sodium excretion, as well as serum creatinine and 24-h magnesium excretion, as their *P*-value was <.1 in univariate analysis. After adjustment, diabetes mellitus (OR 2.94, 95% CI 1.05–8.26, *P* = .041), family history of kidney stones (OR 10.32, 95% CI 4.72–22.53, *P* < .01), and serum creatinine (OR 1.02 per µmol/L, 95% CI 1.00–1.04, *P* = .021) remained independently associated with kidney stone prevalence.

When replacing serum creatinine by eGFR as estimated with the Chronic Kidney Disease Epidemiology Collaboration 2009 formula, similar results were obtained (OR for stone prevalence per mL/min 0.97, 95% CI 0.95–0.99, *P* = .012); hence, a higher eGFR is associated with a lower risk of stone prevalence at baseline.

Other factors—including age, sex, hypertension and urinary parameters—were not associated with stone prevalence (Table 3).

**Table 3:** Univariable and multivariable logistic regression for risk factors associated with kidney stone point prevalence.

	Univariable		Multivariable	
Variable	OR (95% CI)	*P*-value	OR (95% CI)	*P*-value
Age (years)	1.01 (0.99–1.03)	.27	1.01 (0.99–1.03)	.39
Sex (male vs female)	1.28 (0.63–2.60)	.50	1.31 (0.62–2.64)	.51
BMI (kg/m²)	0.94 (0.85–1.04)	.23	0.98 (0.91–1.05)	.49
Education level (per category increase)	0.93 (0.69–1.24)	.60	0.92 (0.69–1.23)	.58
Smoking (current vs never)	1.02 (0.65–1.61)	.88		
Physical activity score (1–10)	0.98 (0.84–1.15)	.84		
Hypertension	1.45 (0.72–2.95)	.30	1.50 (0.74–3.04)	.26
Diabetes mellitus	2.81 (1.01–7.81)	.05	2.94 (1.04–8.26)	.**041**
Personal history of kidney stones	1.95 (0.57–6.65)	.29	2.74 (0.71–10.5)	.14
Family history of kidney stones	9.89 (4.52–21.61)	<.001	10.3 (4.72–22.5)	**<.01**
Serum corrected calcium (mmol/L)	0.50 (0.15–1.65)	.27		
Serum total calcium (mmol/L)	1.07 (0.14–8.11)	.95		
Serum uric acid (µmol/L)	1.00 (0.99–1.00)	.25		
Serum creatinine (µmol/L)	1.02 (1.00–1.04)	.02	1.03 (1.00–1.04)	.**021**
24 h urine volume (mL)	1.00 (0.99–1.00)	.15	1.00 (0.99–1.00)	.18
Urinary calcium (24 h, mmol)	0.96 (0.87–1.07)	.51		
Urinary sodium (24 h, mmol)	1.00 (0.99–1.01)	.64	1.00 (0.99–1.01)	.58
Urinary magnesium (24 h, mmol)	1.19 (1.00–1.42)	.06	0.94 (0.75–1.17)	.56
Urinary phosphate (24 h, mmol)	1.02 (0.99–1.05)	.18		
Urinary uric acid (24 h, mmol)	1.00 (0.99–1.00)	.14		
Urinary pH (daytime)	1.16 (0.95–1.41)	.34		

Values are expressed as ORs with 95% CIs and *P*-values.

Statistical significance was defined as *P* < .05 (in bold).

### Stone incidence after 3 years follow-up

Over a median 3-year follow-up period, 49 new kidney stone cases were identified, corresponding to an incidence of 4.3% (4.1% in men and 4.6% in women). Among these, 39 occurred in participants who were stone-free at baseline, and 2 in participants with stones detected at baseline ultrasound. In addition, eight participants with a prior self-reported history of kidney stones but no stones detected at baseline developed new stones during follow-up. Altogether, these groups accounted for the total of 49 incident cases. Among the 28 participants with a personal history of kidney stones, 11 (39.2%) had developed new stones 3 years later.

In univariable logistic regression, active smoking (OR 2.46, 95% CI 1.04–5.82, *P* = .04), lower physical activity score (OR 0.81 per point increase, 95% CI 0.66–1.00, *P* = .05) and personal history of kidney stones (OR 37.4, 95% CI 10.9–128, *P* < .01) were significantly associated with new stone formation during follow-up. All other demographic, clinical and laboratory variables—including age, sex, BMI, hypertension, diabetes, eGFR and urinary parameters—were not associated with stone incidence.

In the multivariable model adjusted for the same predefined variables as above as well as those with a *P* < .01 in univariate analysis, active smoking (OR 2.64, 95% CI 1.11–6.30, *P* = .028), lower physical activity score (OR 0.78 per point increase, 95% CI 0.64–0.96, *P* = .021) and personal history of kidney stones (OR 36.5, 95% CI 12.9–102.7, *P* < .01) were independent predictors of incident new stones (Table 4).

**Table 4:** Univariable and multivariable logistic regression for risk factors associated with 3-year incidence.

	Univariable		Multivariable	
Variable	OR (95% CI)	*P*-value	OR (95% CI)	*P*-value
Age (years)	0.98 (0.96–1.00)	.11	0.99 (0.97–1.02)	.60
Sex (male vs female)	1.50 (0.73–3.09)	.27	1.52 (0.61–3.77)	.89
BMI (kg/m²)	1.03 (0.95–1.12)	.46	1.04 (0.95–1.13)	.38
Education level (per category increase)	1.05 (0.76–1.45)	.77	0.86 (0.59–1.24)	.41
Smoking (current vs never)	2.46 (1.04–5.82)	.04	2.65 (1.11–6.30)	.**03**
Physical activity score (1–10)	0.81 (0.66–1.00)	.05	0.78 (0.64–0.96)	.**02**
Hypertension	0.59 (0.26–1.33)	.22	0.49 (0.17–1.43)	.19
Diabetes mellitus	(Unstable estimate)	.99		
Personal history of kidney stones	37.40 (10.93–127.99)	<.01	36.5 (12.93–102.6)	**<.01**
Family history of kidney stones	1.27 (0.24–6.65)	.78	1.21 (0.24–6.00)	.82
Serum corrected calcium (mmol/L)	0.96 (0.13–6.90)	.97		
Serum total calcium (mmol/L)	0.70 (0.09–5.45)	.74		
Serum uric acid (µmol/L)	1.00 (0.99–1.01)	.96		
Serum creatinine (µmol/L)	1.01 (0.99–1.03)	.46	1.00 (0.97–1.03)	.91
24-h urine volume (mL)	1.00 (0.99–1.01)	.78	1.00 (0.99–1.00)	.06
Urinary calcium (24 h, mmol)	0.91 (0.82–1.06)	.15		
Urinary sodium (24 h, mmol)	0.99 (0.99–1.01)	.76	1.00 (0.99–1.01)	.13
Urinary magnesium (24 h, mmol)	0.92 (0.83–1.01)	.06		
Urinary phosphate (24 h, mmol)	0.98 (0.95–1.05)	.18		
Urinary uric acid (24 h, mmol)	1.00 (0.99–1.00)	.14		
Urinary pH (daytime)	0.98 (0.95–1.05)	.75		

Values are expressed as OR swith 95% CIs and *P*-values.

Statistical significance was defined as *P* < .05 (in bold).

Unstable estimate: excluded from multivariable model due to data instability (none of the patients with DM had developed stones at follow-up).

## Discussion

This population-based study (SKIPOGH) provides updated epidemiological insights into the prevalence and incidence of urolithiasis in Switzerland. The point prevalence of kidney stones was 5.6%, with comparable rates in men (6.1%) and women (5.1%), aligning with the few previously reported European estimates of 5%–9% and global prevalence patterns [1, 2]. The 3-year incidence of new kidney stones was 4.3%, underscoring the persistent risk of stone formation in the adult general population. This finding aligns with previous longitudinal studies that have demonstrated the recurrent nature of urolithiasis and its tendency to reappear within a few years of initial diagnosis [6, 9], emphasizing the importance of long-term monitoring and preventive strategies in at-risk individuals.

Risk factors for prevalent kidney stones in our cohort included diabetes mellitus, a family history of kidney stones and higher serum creatinine. Family history of nephrolithiasis was one of the strongest predictors of prevalent kidney stones, with individuals having affected relatives showing 10-fold higher odds of stone presence at baseline. The result for diabetes mellitus is consistent with previous studies, highlighting the metabolic and renal vulnerability of stone formers [3, 9]. Diabetes contributes to stone formation through multiple mechanisms, including reduced urinary pH, increased urinary calcium and uric acid excretion, and alterations in renal handling of solutes—conditions that favor the formation of both uric acid– and calcium-based stones [3, 14].

The positive association between kidney stone prevalence and serum creatinine should be interpreted with caution. In cross-sectional analysis, elevated serum creatinine, as a marker of reduced kidney function, may reflect either a consequence of nephrolithiasis or a shared pathophysiologic pathway. Nevertheless, impaired renal function is associated with altered urine composition, such as decreased citrate and increased phosphorus concentrations, which can both promote lithogenesis [15, 16]. Besides, kidney stone prevalence also correlated with estimated GFR: the lower eGFR; the higher the risk of stone prevalence. While both creatinine and eGFR reached statistical significance, the effect size was very small, and we cannot exclude residual confounding factors such as muscle mass, rather than a true causal relationship.

In contrast to earlier research, traditional cardiovascular risk factors such as hypertension and obesity were not independently associated with prevalent kidney stones in our multivariable analysis [5]. This divergence may reflect differences in study populations, including age distribution and underlying comorbidities, as well as variations in diagnostic criteria, statistical adjustments and definitions of exposure. It is also possible that the influence of these risk factors is mediated through other metabolic pathways—such as insulin resistance or altered urine composition—that were not fully captured in our dataset [10].

As for the 3-year incidence analysis, the most striking result was the strong association between new kidney stone formation and a prior personal history of stones, with almost half of participants with a personal history of stones developing new stones at follow-up. The extremely high OR observed for personal history underscores its strong predictive value for recurrence risk. Interestingly, family history of kidney stones was associated with prevalent stones but not with incident stones. One possible explanation is that individuals with a family history of nephrolithiasis may have an inherited or shared environmental predisposition that promotes early disease onset, leading to higher prevalence but not necessarily new stone formation during follow-up. In contrast, personal history reflects both prior exposure and individual recurrence risk, explaining its stronger link with incident stones.

Other factors associated with stone incidence were active smoking and a lower physical activity score. The link between active smoking and incident stone formation aligns with prior evidence suggesting increased urinary oxidative stress, altered calcium handling and dehydration-related mechanisms in smokers [16, 17]. The inverse association with physical activity is of interest, and in line with previous studies. Low physical activity predisposes to metabolic disturbances, obesity and diabetes mellitus, and a high daily sitting time has been linked to stone risk when not compensated by vigorous recreational activity [18] Inversely, high physical activity is typically protective against cardiometabolic disease. Further studies are needed to clarify whether this finding reflects a true physiological effect, behavioral patterns (e.g. exercise with inadequate hydration) or unmeasured confounding.

No significant associations were observed between incident kidney stone formation and 24-h urinary excretion of key electrolytes, including calcium, sodium and uric acid. Although these urinary parameters are central to individualized stone prevention and metabolic evaluation, their predictive value in population-based models of stone incidence appears limited [19]. This finding is consistent with prior cohort studies, which suggest that while biochemical abnormalities in urine may contribute to pathophysiology, they do not reliably predict new stone events at the population level [9, 20].

This study’s strengths include its multicenter, population-based design, standardized ultrasound-based assessment of nephrolithiasis, and comprehensive evaluation of metabolic, urinary and behavioral risk factors. The use of trained sonographers across all centers ensured consistency in stone detection, while the rich dataset allowed for robust multivariable analyses.

Nonetheless, several limitations must be acknowledged. First, renal ultrasound, although non-invasive and widely available, is less sensitive than computed tomography for detecting kidney stones, which is considered the reference standard for stone detection. As a result, the point prevalence reported in this study likely represents a conservative, lower-bound estimate of the true prevalence of urolithiasis. In particular, small stones (especially <3 mm), non-obstructive calculi, lower-pole renal stones and ureteral stones are more likely to be missed by ultrasonography, potentially leading to underdiagnosis [21]. Second, the reliance on self-reported history for lifetime stone events may introduce recall bias. Lastly, the observed associations between smoking, physical activity and incident stones may reflect unmeasured confounders such as hydration status, dietary intake or exercise intensity, and warrant further investigation.

## Conclusion

Our findings highlight both the metabolic and hereditary components of kidney stone disease, identifying diabetes mellitus, family history of kidney stones and higher serum creatinine as significant risk factors for stone prevalence. Moreover, a personal history of kidney stones, active smoking and lower physical activity levels emerged as the main predictors of new stone formation over 3 years, with the very high OR for personal history emphasizing its strong link to recurrence risk. These results underscore the importance of integrating both personal and familial history into routine risk assessments and reinforce the need for individualized preventive strategies—particularly promoting healthy lifestyle habits—to mitigate recurrence and incident risk in susceptible individuals.

## Data Availability

The data underlying this article will be shared on reasonable request to the corresponding author.
